# Cognitive training and promoting a healthy lifestyle in isolated REM sleep behavior disorder: The randomized controlled trial CogTrAiL‐RBD

**DOI:** 10.1002/alz.71707

**Published:** 2026-08-05

**Authors:** Anja Ophey, Sinah Röttgen, Konstantin Kufer, Julia Pauquet, Daniel Scharfenberg, Philipp Armbrust, Anastasia Kammerzell, Nathalie Knopf, Sandy Kollath, Philipp Sommer, Christopher E. J. Doppler, Ezequiel Farrher, Aline Seger, N. Jon Shah, David Zopfs, Kathrin Reetz, Gereon R. Fink, Michael Sommerauer, Elke Kalbe

**Affiliations:** ^1^ Department of Medical Psychology | Neuropsychology and Gender Studies, Center for Neuropsychological Diagnostics and Intervention (CeNDI), Faculty of Medicine and University Hospital Cologne University of Cologne Cologne Germany; ^2^ Cognitive Neuroscience Institute for Neuroscience and Medicine 3 (INM‐3) Research Centre Juelich Juelich Germany; ^3^ Department of Neurology Faculty of Medicine and University Hospital Cologne University of Cologne Cologne Germany; ^4^ Department of Parkinson, Sleep and Movement Disorders, Center for Neurology University Hospital Bonn University of Bonn Bonn Germany; ^5^ German Centre for Neurodegenerative Diseases (DZNE) Bonn Germany; ^6^ Institute for Neuroscience and Medicine ‐ Imaging Core Facility (INM‐ICF) Research Centre Juelich Juelich Germany; ^7^ Institute for Neuroscience and Medicine 4 (INM‐4) Research Centre Juelich Juelich Germany; ^8^ Department of Neurology RWTH Aachen University Aachen Germany; ^9^ JARA‐BRAIN,Translational Medicine Aachen Germany; ^10^ Institute for Neuroscience and Medicine 11 (INM‐11) Research Centre Juelich Juelich Germany; ^11^ Institute for Diagnostic and Interventional Radiology Faculty of Medicine and University Hospital Cologne University of Cologne Cologne Germany; ^12^ JARA‐BRAIN Institute Molecular Neuroscience and Neuroimaging Research Centre Juelich Juelich Germany

**Keywords:** brain health, cognitive training, lifestyle intervention, prodromal Parkinson's disease, REM sleep behavior disorder, secondary dementia prevention

## Abstract

**INTRODUCTION:**

Isolated rapid eye movement (REM) sleep behavior disorder (iRBD) is a prodromal phase of Lewy body diseases and linked to a high risk for cognitive decline. Therefore, iRBD provides a suitable window for early intervention.

**METHODS:**

In the monocentric randomized controlled trial “**Cog**nitive **Tr**aining & a Healthy, **A**ct**i**ve **L**ifestyle Program for People with Isolated **R**EM Sleep **B**ehaviour **D**isorder” (CogTrAiL‐RBD), the feasibility and behavioral effects of a 5‐week digital, adaptive, multidomain cognitive training alongside a module promoting a healthy lifestyle (INT) were compared to a waiting‐list control group (CON) in 82 individuals with polysomnography‐confirmed iRBD. The primary outcome was change in executive functions at 6 weeks post‐allocation (t1) and at 6‐month follow‐up (t2).

**RESULTS:**

For executive functions, we found positive intervention effects for the INT versus CON with a medium effect at t1 (*d *= 0.500, 95% confidence interval [CI]: 0.221–0.779) and a small effect at t2 (*d *= 0.397, 95% CI: 0.115–0.679).

**DISCUSSION:**

CogTrAiL‐RBD provides initial evidence for the feasibility and potential cognitive benefits of cognitive training within a lifestyle‐guidance framework in individuals with iRBD. Its suitability for dementia prevention warrants further investigation.

## Highlights

- People with isolated rapid eye movement (REM) sleep behavior disorder (iRBD) are at risk for cognitive decline.
- **Cog**nitive **Tr**aining & a Healthy, **A**ct**i**ve **L**ifestyle Program for People with Isolated **R**EM Sleep **B**ehaviour **D**isorder (CogTrAiL‐RBD) combines digital cognitive training and a lifestyle module for iRBD.
- Non‐pharmacological cognitive enhancement in iRBD is feasible and effective.
- Secondary dementia prevention in prodromal Lewy body diseases warrants further investigation.

## 1 BACKGROUND

The *2024 Lancet Commission Report on Dementia Prevention* estimated that 14 modifiable risk factors account for almost 45% of dementia cases worldwide.^1^ Over the past decade, large‐scale randomized controlled trials on dementia prevention have been conducted in populations at risk for Alzheimer's disease and related dementias. The Finnish Geriatric Intervention Study to Prevent Cognitive Impairment and Disability (FINGER) was the first to demonstrate that a 2‐year multidomain lifestyle intervention—combining cognitive training, physical activity, nutritional counseling, and vascular risk monitoring—can preserve cognitive function in older adults at risk for dementia.^2^ Although the results of other large‐scale multidomain lifestyle intervention trials are mixed,^3^, ^4^, ^5^, ^6^, ^7^ the U.S. Study to Protect Brain Health Through Lifestyle Intervention to Reduce Risk (U.S. POINTER) demonstrated that FINGER's results generalize to a larger, more diverse U.S. population.^8^ Furthermore, targeted short‐term cognitive training alone has been shown to produce durable improvements in trained abilities and to help maintain daily functioning over a decade.^9^, ^10^ Meta‐analytical evidence supports the feasibility and efficacy of cognitive training to enhance cognition in older adults.^11^, ^12^ Together these studies underscored a paradigm shift and established a blueprint for prevention trials grounded in modifiable risk reduction and cognitive enhancement for those at‐risk for dementia to maintain cognitive health and delay clinical progression.^3^, ^9^

The potential and necessity of dementia prevention has also been recognized for Lewy body diseases (LBDs), including Parkinson's disease (PD).^13^ Cognitive decline is a frequent and debilitating non‐motor symptom for people living with PD,^14^ and up to 74% of individuals develop dementia within 20 years of PD diagnosis.^15^ The relevance of lifestyle interventions for symptom management in PD has recently been emphasized in a comprehensive review.^16^ To date, the strongest evidence for positive effects on both motor and non‐motor domains and potential disease‐modifying effects in PD has been reported for physical activity, particularly moderate‐ to high‐intensity aerobic exercise.^16^ However, interventional evidence in non‐PD LBD remains sparse and, of note, the cognitive domain is overall strongly underrepresented—both as an outcome and as an intervention target.^16^, ^17^

Meta‐analyses have already demonstrated that cognitive training can yield measurable and lasting improvements in cognitive performance among people living with PD.^18^, ^19^, ^20^ Digital cognitive training approaches are usually administered over short periods of 5–6 weeks, with most studies evaluating adaptive, multi‐domain cognitive training. Effects tend to be small for global cognition, but may be more pronounced for higher‐order cognitive functions such as fluid reasoning.^20^ Potential mechanisms underlying cognitive training responsiveness may include neuroplastic changes and enhanced compensatory processes, potentially contributing to cognitive reserve and resilience against neurodegeneration.^21^

A unique opportunity for secondary dementia prevention in the context of LBDs arises in rapid eye movement (REM) sleep behavior disorder (RBD), a parasomnia characterized by loss of muscle atonia and dream enactment during REM sleep.^22^ Approximately 80%–90% of individuals with isolated or idiopathic RBD (iRBD) show seeding competent α‐synuclein species in skin or cerebrospinal fluid, illustrating iRBD biologically as an early α‐synucleinopathy.^23^, ^24^, ^25^, ^26^, ^27^ Accordingly, up to 90% of individuals with iRBD will eventually phenoconvert to clinically manifest PD or dementia with Lewy bodies within 15 years after diagnosis, with almost similar probability and an annual rate of ≈6%.^24^, ^28^ Cross‐sectional studies consistently indicate that individuals with iRBD perform worse than healthy controls across multiple cognitive domains.^29^ Approximately 25%–30% of individuals with iRBD already meet criteria for mild cognitive impairment (MCI),^29^, ^30^ with its presence tripling the risk of subsequent phenoconversion.^29^ iRBD thus represents a predictable pre‐diagnostic phase, analogous to the “at‐risk” cohorts targeted in Alzheimer's prevention research, and provides a well‐defined window for implementing neuroprotective and cognition‐preserving interventions.

With the **Cog**nitive **Tr**aining & a Healthy, **A**ct**i**ve **L**ifestyle Program for People with Isolated **R**EM Sleep **B**ehaviour **D**isorder (CogTrAiL‐RBD)^31^ study, we aim to investigate the feasibility and short‐ and long‐term efficacy of a multidomain cognitive training intervention within a lifestyle‐guidance framework for individuals with iRBD, with change in executive functions as the primary outcome.

**RESEARCH IN CONTEXT**

1. **Systematic review**: We reviewed evidence from randomized trials and meta‐analyses identified through databases including MEDLINE and Cochrane. Multidomain lifestyle interventions and cognitive training interventions show small but consistent cognitive benefits in at‐risk populations. However, no randomized controlled trials have evaluated cognitive training or multidomain lifestyle interventions in isolated rapid eye movement (REM) sleep behavior disorder (iRBD), a possible prodromal phase of Parkinson's disease and dementia with Lewy bodies.
2. **Interpretation**: This randomized trial demonstrates that a digital, adaptive, multidomain cognitive training within a lifestyle‐guidance framework may improve executive functions in iRBD, with sustained effects at 6 months. These findings extend prior work to a prodromal population and support further investigation of scalable non‐pharmacological strategies for early secondary dementia prevention.
3. **Future directions**: Future research should confirm these findings in larger, multicenter randomized controlled trials with longer follow‐up to determine the durability of cognitive benefits and potential effects on phenoconversion to Parkinson's disease or dementia.

## 2 METHODS

### 2.1 Study design

The CogTrAiL‐RBD study was initiated at the University Hospital Cologne, Germany, and designed as a monocentric, single‐blind, randomized controlled trial with two study arms and an exploratory delayed‐start, open‐label trial extension. Here, we report the behavioral results from the parallel‐group phase (superiority design) across the first three assessment timepoints: baseline (t0), the 6‐week post‐allocation visit (t1), and the 6‐month follow‐up (t2).

Participants were randomized to either the intervention group (INT) or a passive waiting‐list control group (CON). The INT received a 5‐week digital cognitive training alongside a module promoting a healthy, active lifestyle, following the t0 baseline assessment. The parallel‐group phase of CogTrAiL‐RBD has been completed on September 5, 2025, and the exploratory open‐label phase reached the last‐patient‐last‐visit on April 24, 2026. The full study protocol has been published previously.^31^ In addition to the mandatory clinical and neuropsychological assessments, optional, exploratory study modules included accelerometry and magnetic resonance imaging (MRI) conducted at the Research Centre Juelich, Germany. Exploratory accelerometry as well as structural and functional MRI outcomes will be analyzed and reported elsewhere.

Ethical approval was obtained from the Ethics Committee of the Medical Faculty of the University of Cologne (2022‐03‐09; Identifier: 21‐1291). The trial was prospectively registered in the German Clinical Trials Register (2022‐03‐11, Identifier: DRKS00024898).

### 2.2 Participants

The following inclusion criteria were applied: (1) polysomnography‐proven diagnosis of iRBD, (2) age 40–80 years, (3) normal/corrected‐to‐normal vision and hearing, (4) sufficient proficiency in German, and (5) access to a local computer with access to the internet. Exclusion criteria were: (i) severe cognitive dysfunction (Montréal Cognitive Assessment^32^ [MoCA], ≤22), (2) significant neurological and psychiatric concomitant diseases, and, for those willing to participate in the optional MRI module, (3) contraindications for MRI.

Participants were consecutively recruited from continuously expanding local iRBD cohorts in the Rhineland^33^, ^34^ (Cologne, Bonn, Aachen; Germany). During active recruitment for the local iRBD cohorts, individuals were informed prior to screening that iRBD is associated with an estimated ≈80% risk of progression to a neurodegenerative disorder such as PD, thereby ensuring the possibility of withdrawing before data collection—including polysomnography‐confirmation of RBD, informed decision‐making, and autonomy in the risk disclosure process from the outset.^33^, ^35^, ^36^ Following confirmation of iRBD, participants were informed about their diagnosis, educated once again regarding its implications, and invited to participate in annual clinical follow‐ups within the cohort framework.^33^ Individuals interested in CogTrAiL‐RBD study participation were assessed for eligibility at the University Hospital Cologne between June 14, 2022, and December 13, 2024. Written informed consent to participate was obtained from all participants.

### 2.3 Randomization and masking

Participants were randomized to one of the two groups (INT vs CON) in a 1:1 ratio. Randomization was performed using a computerized minimization procedure implemented in the R package *Minirand* ^37^ to balance groups across three equally weighted covariates (age, sex, premorbid intelligence quotient [IQ]).^38^ No other covariates were used for stratification. Allocation concealment was ensured through central randomization in R, accessible only to authorized study personnel. Participants were assigned to their respective group following the t0 assessment by a staff member not involved in subsequent data collection. This person also oversaw the intervention and served as the main contact for participants throughout the study. Assessments were conducted under single‐, experimenter‐blinded conditions at post‐intervention timepoints. Any violations of blinding were documented.

### 2.4 Procedures

#### 2.4.1 Cognitive training intervention

The cognitive training intervention consisted of a 5‐week, digital, adaptive, multidomain cognitive training combined with a module promoting a healthy, active lifestyle. The latter included psychoeducational information and activity monitoring via a digital activity diary. The intervention duration was aligned with previously published digital cognitive training interventions.^9^, ^10^, ^20^ This rather short intervention—compared to the longer‐term dementia prevention frameworks such as FINGER^2^—was chosen because the primary aim of this study was to assess feasibility and the potential to induce cognitive plasticity via cognitive training in individuals with iRBD, conceptualized as an analogous at‐risk cohort to those targeted in Alzheimer's disease and all‐cause dementia prevention research. Of note, in contrast to long‐term multidomain prevention trials, the present intervention did not primarily aim to modify lifestyle risk factors comprehensively but focused on short‐term effects and feasibility in a prodromal population.

The cognitive training was delivered using *HeadApp/NEUROvitalis Digital* (HelferApp GmbH, Gommern, Germany), which provides remotely accessible, digital, adaptive, multidomain cognitive training. The training plan comprised 15 sessions over 5 weeks (three sessions per week, four 10‐min tasks, i.e., 40 min per session), resulting in a maximum total training duration of 600 minutes. Participants were instructed to distribute training sessions evenly across the interval. Only one training session per day was technically enabled by the platform. The program included six tasks, each with a different cognitive focus (executive functions, working memory, episodic memory, attention, visuo‐cognition, and language). Training difficulty adapted to participants’ performance. With higher difficulty levels, all tasks involved a strong executive component. The training plan and platform access were introduced to participants by trained psychologists following randomization. Participants completed the training independently, with individual support available via telephone or email for queries. Training adherence was tracked via the HeadApp platform.

The module promoting a healthy, active lifestyle comprised psychoeducational content and activity self‐monitoring. Psychoeducational topics included risk and protective factors for healthy aging; the concepts of cognitive and motor reserve; strategies for incorporating cognitive, social, and physical activities into daily routines; and the role of the Mediterranean diet for cognitive health. The material was provided in a graphic booklet adapted from the scientifically validated NEUROvitalis program (ProLog, Therapie und Lernmittel GmbH, Cologne, Germany) and is available in German upon request from the corresponding authors. During the parallel‐group phase, participants of the INT additionally completed digital activity diaries weekly (t0‐t1) and monthly (t1–t2), documenting physical activity with the short version of the International Physical Activity Questionnaire (IPAQ),^39^, ^40^ cognitive and social lifestyle activities with the Lifestyle Activities Questionnaire,^41^, ^42^ and Mediterranean diet adherence with the Mediterranean Diet Adherence Screener (MEDAS).^43^, ^44^ This self‐monitoring aimed to foster reflection and encourage the adoption of active routines. Please note that the module promoting a healthy, active lifestyle did not contain face‐to‐face, personalized health counseling or an extended multidomain intervention package addressing lifestyle domains beyond cognitive training (e.g., supervised/guided physical activity, nutritional counseling, or psychotherapy) as implemented in the large‐scale multidomain dementia prevention trials.^2^, ^3^, ^4^, ^5^, ^6^, ^7^, ^8^ Therefore, the module is most comparable to the educational component used in the self‐guided control condition of the U.S. POINTER trial.^8^

There was no systematic personal contact between participants and the study team during the intervention phase. Personal contact was established only if problems arose with either the digital cognitive training or the digital activity diary.

#### 2.4.2 Study visits

Study visits were scheduled via telephone. All participants, regardless of group assignment, received a standardized email with a calendar reminder prior to each study visit. In‐person study visits t0–t2 included paper–pencil cognitive testing and motor evaluations. For cognitive testing, parallel test forms alternating between study visits were used if available. Outcome assessors were psychologists, neuroscientists, or trained graduate students of psychology or medicine at all timepoints. MCI status was assessed according to the Movement Disorder Society (MDS) Level‐II criteria for MCI in PD^45^ including subtype‐specifications based on the involvement of memory impairment (amnestic “a” vs non‐amnestic “na”) and the number of affected domains (single‐domain “sd” vs multi‐domain “md”), as described previously.^30^ After completion of the cognitive test battery, the Purdue Pegboard test^46^, ^47^ was used to assess fine motor skills, and part three of the MDS Unified Parkinson's Disease Rating Scale (MDS‐UPDRS‐III^48^) was used to assess PD‐related motor symptoms. Following the in‐person assessments, participants completed digital questionnaires on non‐motor symptoms, quality of life, and lifestyle activities. Cognitive reserve was assessed with the Lifetime of Experiences Questionnaire (LEQ).^49^ A complete list of all cognitive assessments, motor evaluations, and questionnaires, including references and an indication of the use of parallel test forms, is published in the study protocol.^31^ Data on medication use and olfactory function were obtained from time‐matched clinical visits.

Brain imaging was conducted at the Research Centre Juelich with a 3T SIEMENS PRISMA scanner equipped with a 64‐channel head coil. Details on the T1 sequence parameters and FreeSurfer‐based preprocessing for extracting cortical thickness (whole‐brain and Desikan–Killiany atlas‐based) are reported in Supplementary Material 1.

### 2.5 Outcomes

#### 2.5.1 Feasibility outcomes

Feasibility outcomes include the participation rate, compliance, and the overall dropout rate across timepoints, compared between groups. The participation rate was defined as the proportion of individuals with iRBD who agreed to participate relative to the number initially contacted. Compliance was operationalized as adherence to the intervention protocol, assessed by (1) successful completion of at least 80% of planned cognitive training sessions (≥12 sessions), and (2) consistent use of the digital activity diary (≥3/5 of weekly questionnaires, ≥4/6 of monthly questionnaires).

#### 2.5.2 Primary, secondary, and exploratory outcomes

Intervention effects are evaluated as the change from t0 to the respective post‐allocation timepoints, i.e., t1 for short‐term effects and t2 for long‐term effects. The primary outcome is an equally weighted composite score of executive functions comprising subscores of logical reasoning, set‐shifting, semantic and phonemic verbal fluency, and interference control. In addition to reporting the analyses based on the raw executive composite score, we report robust and distribution‐based clinically meaningful improvement (robust CMI, rCMI^50^, ^51^; for details, see Supplementary Material 2).

Secondary cognitive outcomes include equally weighted composite scores for other cognitive domains (attention & working memory, memory, visuo‐cognition, and language), as well as a global cognition composite score derived from these domain scores. The single cognitive test scores were demographically adjusted and standardized using published normative data, and transformed into *z*‐scores during preprocessing. Single cognitive test scores and the participant's MCI status served as exploratory cognitive outcomes.

Additional secondary participant‐reported outcome measures (PROMs) comprise subjective cognitive decline (Multiple domain Subjective Cognitive Decline Evaluation, Multi‐SubCoDE SCD‐Severity^30^), depressive symptoms (Beck Depression Inventory, BDI‐II total score^52^), fatigue (Fatigue Scale for Motor and Cognitive Functions, FSMCF total score^53^), and health‐related quality of life (Short‐Form 36, SF‐36 total score^54^, ^55^). Secondary motor outcomes include PD‐related motor symptoms (MDS‐UPDRS‐III total score^48^) and fine motor dexterity (Purdue Pegboard both‐hands and assembly^46^, ^47^).

### 2.6 Statistical analyses

An a priori power analysis was conducted[Fig alz71707-fig-0001] with G*Power (http://www.gpower.hhu.de55) to determine the target sample size for detecting moderate behavioral effects in executive functions (primary outcome) in the first parallel‐group phase of CogTrAiL‐RBD. Based on a medium effect size (Cohen's *d *= 0.5), α = 0.05, 80% power, and accounting for anticipated high baseline–retest correlations in the executive function composite score, the minimum required sample was *N *= 76 (*n *= 38 per group). Allowing for a 5% dropout, the final recruitment target for CogTrAiL‐RBD was *N *= 80. Full details are provided in the study protocol.^31^ Given the exploratory proof‐of‐concept design and recruitment challenges associated with polysomnography‐confirmed iRBD, a descriptive interim evaluation of recruitment progress, adherence, and data quality was conducted during the study; no formal stopping rules were predefined.

Data analysis was performed in R (version 4.5.0) with *ggplot2* (version 3.5.0) and *ggseg* (version 1.6.5) for data visualization. Baseline characteristics were compared between groups using independent samples *t*‐tests or Wilcoxon rank‐sum tests for continuous variables (depending on normality as assessed by Shapiro–Wilk tests), and chi‐square tests for categorical variables. Training effects on continuous primary and secondary outcomes were analyzed using linear mixed‐effects (LME) models with time (t0, t1, t2), group (CON = 0 vs INT = 1), and their interaction (time*group) as fixed effects, and participants as random intercepts. No additional covariates were included in the models.

Models were estimated using the *lme4*‐package employing restricted maximum likelihood estimation. The significance of main effects (time, group) and their interaction was assessed using Type‐III analysis of variance (ANOVA) with Satterthwaite's approximation for denominator degrees of freedom. For interaction effects, Cohen's *d* with 95% confidence intervals (95% CIs) is reported. In addition, group‐wise change scores for each timepoint based on estimated marginal means are reported. Statistical significance was set at a[Table alz71707-tbl-0001] two‐sided α‐level of 0.05. Training‐related group differences in dichotomous outcomes (rCMI, MCI) were examined using logistic regression models. Odds ratios (ORs) and corresponding 95% CIs were reported as effect sizes. The significance level was adjusted using the Benjamini–Hochberg procedure, controlling the false discovery rate (FDR) across multiple comparisons. Analyses followed the intention‐to‐treat principle, including all randomized participants. No imputation methods were used.

Exploratory analyses evaluated predictors of intervention responsiveness with multiple linear regression models. The absolute change in the primary outcome (Delta*_t_*_1‐t0_ or Delta*_t_*_2–_ *_t_*_0_) was used as the dependent variable. Age, executive performance, global cognition, cognitive reserve, and, if available, mean cortical thickness—all assessed at t0—were entered as independent variables. Separate models were fit for each group, timepoint, and according to MRI availability. Coefficient uncertainty was assessed using nonparametric bootstrapping with 5000 resamples and bias‐corrected and accelerated (BCa) 95% CIs.

## 3 RESULTS

### 3.1 Flow of participants, feasibility, and sample characteristics

**FIGURE 1 alz71707-fig-0001:**
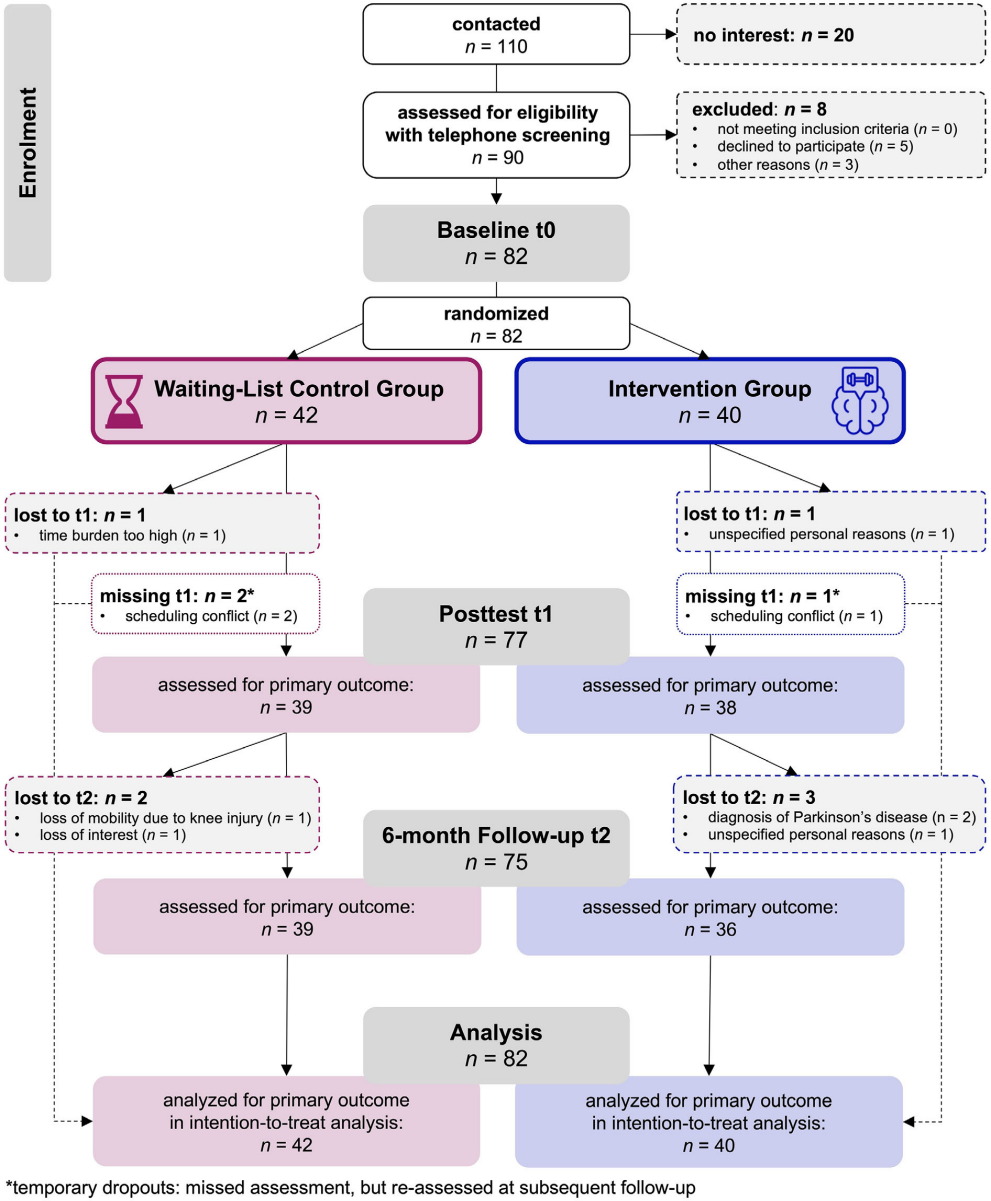
Flow of participants through the **Cog**nitive **Tr**aining & a Healthy, **A**ct**i**ve **L**ifestyle Program for People with Isolated **R**EM Sleep **B**ehaviour **D**isorder (CogTrAiL‐RBD) trial.

Figure 1 shows the flow of participants through each stage of the trial. Of 110 individuals initially interested in CogTrAiL‐RBD, 20 withdrew after receiving more detailed information. Telephone screening was conducted with 90 individuals with iRBD. The main reasons for declining participation after the telephone screening were personal time constraints and the study‐related (time) burden, including multiple on‐site visits. A total of 82 individuals with iRBD were eligible, provided written informed consent for study participation, completed the t0 assessment, and were subsequently randomized to the CON (*n *= 42) or the INT (*n *= 40), resulting in a participation rate of 74.5% (82/110).

Between t0 and t1, one participant in each group was lost to follow‐up, and three additional t1 assessments were missed due to scheduling conflicts. Accordingly, the dropout rate was 2.4% (2/82) between t0 and t1, or 6.1% (5/82) if temporary dropouts were included. Between t1 and t2, two participants in the CON and three in the INT, of which two received the clinical PD diagnosis, were lost to follow‐up, resulting in an additional dropout rate of 6.1% (5/82). Overall, 97.8% (80/82) of randomized participants completed at least one post‐allocation assessment.

Ninety percent of individuals randomized to the INT (36/40) completed at least 80% of the planned training sessions. Two individuals did not train at all: one was a t1 dropout, and the other was a t1 missing case. The median training duration was 526.3 min (interquartile range [IQR] 497.3–550.2), distributed over 30 days (IQR 28–32) between the first and last training day. No adverse events were reported. The weekly activity diary was maintained in at least 3 of 5 weeks by 82.5% (33/40) of individuals, and the monthly activity diary in at least 4 of 6 months by 86.8% (33/38).

The mean age of participants was 69.24 (SD = 5.90) years of age, and 87.8% (72/82) were male. The mean time since the subjectively reported onset of RBD symptoms was 9.47 (SD = 6.45) years. On average, participants had 15.62 years of education (SD = 3.01). Based on the t0 cognitive assessment, 31.7% (26/82) of individuals with iRBD met the MDS Level‐II criteria for MCI. Group‐wise sample characteristics are reported in Table 1 and Table S1. The two groups were comparable with regard to demographic, clinical, cognitive, and lifestyle‐related characteristics (all *p*’s > 0.05, unadjusted for multiple comparisons).

**TABLE 1 alz71707-tbl-0001:** Sample characteristics.

	Control Group (CON)	Intervention Group (INT)
	*n* = 42	*n* = 40
Age, years	68.88 (6.30)	69.62 (5.50)
	[55.90–80.93]	[57.64–80.31]
Sex, *n* (%)		
Female	5 (11.90%)	5 (12.50%)
Male	37 (88.10%)	35 (87.50%)
Education, years	15.33 (3.04)	15.91 (3.00)
	[7–21]	[10–22]
Time since first reported RBD symptoms, years	7.57 (4.85–11.57)	7.99 (4.30–12.70)
	[1.92–30.93]	[1.97–27.56]
RBDSQ total score^a^	9.00 (7.50–11.00)	9.50 (6.00–11.00)
	[2–12]	[0–13]
MDS‐UPDRS‐III total score	5.00 (3.00–8.00)	5.50 (2.50–9.00)
	[0–19]	[0–23]
Purdue Pegboard both hands^b^	−0.60 (‐1.30, 0.07)	−0.39 (‐0.84, 0.07)
	[‐2.94–4.00]	[‐2.90–1.10]
Purdue Pegboard assembly^b^	−0.04 (0.96)	−0.340 (0.98)
	[‐2.24–2.39]	[‐2.17–1.84]
Sniffin’ Sticks total score^c^	6.36 (2.54)	6.72 (2.46)
	[0–12]	[0–12]
Sniffin’ Sticks category^c^, *n* (%)		
Normosmia (10–16)	3 (8.33%)	6 (16.67%)
Hyposmia (6–9)	22 (61.11%)	22 (61.11%)
Anosmia (0–5)	11 (30.56%)	8 (22.22%)
BDI‐II total score^d^	5.00 (1.00–9.00)	4.00 (1.00–9.00)
	[0–17]	[0–28]
BDI‐II category^d^, *n* (%)		
No depression (0–8)	28 (71.79%)	28 (71.79%)
Minimal depression (9–13)	8 (20.51%)	4 (10.26%)
Mild depression (14–19)	3 (7.69%)	5 (12.82%)
Moderate depression (20–28)	0 (0.00%)	2 (5.13%)
NMSQ total score^d^	5.00 (3.00–8.00)	7.00 (5.00–9.00)
	[0–14]	[0–18]
MoCA total score	26.00 (24.00–27.00)	27.00 (24.00–28.00)
	[23–30]	[23–30]
Level‐II MCI, *n* (%)		
No MCI	27 (64.29%)	29 (72.50%)
MCI	15 (35.71%)	11 (27.50%)
Level‐II MCI subtypes, *n* (%)		
‐ na‐sd‐MCI	1 (2.38%)	0 (0.00%)
‐ a‐sd‐MCI	1 (2.38%)	1 (2.50%)
‐ na‐md‐MCI	4 (9.52%)	5 (12.50%)
‐ a‐md‐MCI	9 (21.43%)	5 (12.50%)
IPAQ total score^d^	4,584.00 (2,466.00–6,477.10)	3,510.00 (2,050.00, 5,580.00)
	[726–15,624]	[0–18,025]
LAQ total score^d^	197.80 (60.81)	196.21 (62.55)
	[60–346]	[66–330]
MEDAS total score^d^	5.231 (2.52)	6.077 (2.18)
	[0–12]	[2–11]

*Note*: Data are mean (SD) [range: minimum—maximum], median (interquartile range) [range: minimum—maximum], or *n* (%). Abbreviations: a‐md‐MCI, amnestic multi‐domain MCI; a‐sd‐MCI, amnestic single‐domain MCI; BDI‐II, Beck Depression Inventory; IPAQ, International Physical Activity Questionnaire; LAQ, Lifestyle Activities Questionnaire; MCI, mild cognitive impairment; MDS‐UPDRS‐III, Movement Disorder Society Unified Parkinson's Disease Rating Scale Part 3; MoCA, Montréal Cognitive Assessment; MEDAS, Mediterranean Diet Adherence Screener; na‐sd‐MCI, non‐amnestic single‐domain MCI; na‐md‐MCI, non‐amnestic multi‐domain MCI; NMSQ, Non‐Motor Symptoms Questionnaire; RBD, rapid eye movement (REM) sleep behavior disorder; RBDSQ, REM Sleep Behavior Disorder Screening Questionnaire.

^a^ Missing for *n* = 2 in the CON, and *n* = 2 in the INT.

^b^ Missing for *n* = 0 in the CON, and *n* = 1 in the INT.

^c^ 16‐item smell test by Burkhart Messtechnik GmbH, Germany; missing for *n* = 6 in the CON, and *n* = 4 in the INT.

^d^ Missing for *n* = 3 in the CON, and *n* = 1 in the INT.

### 3.2 Training effects

**FIGURE 2:**
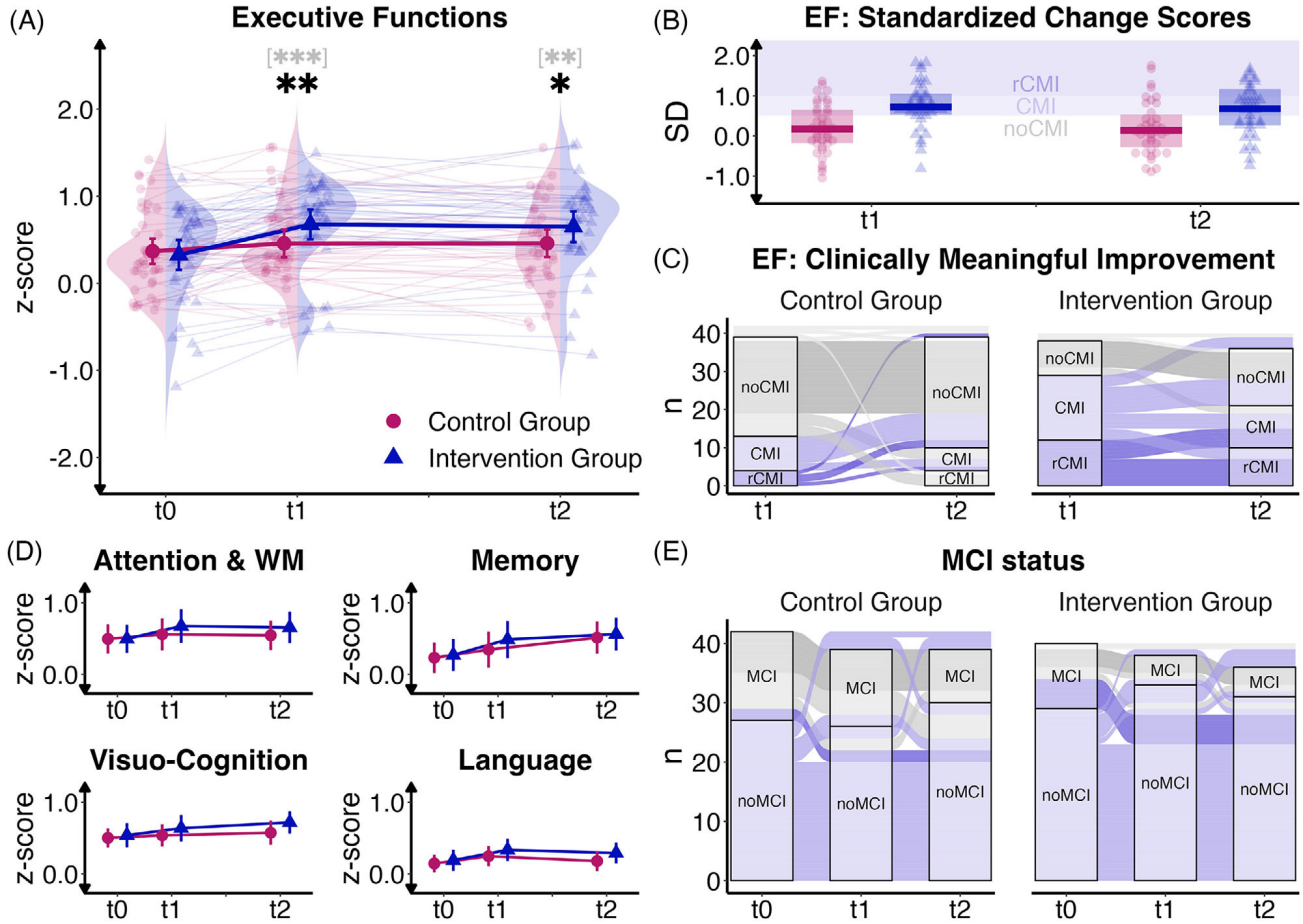
Cognitive Outcomes of CogTrAiL‐RBD. (A) Mean observed composite score of executive functions (EF) across t0 (baseline), t1 (6‐week post‐allocation), and t2 (6‐month follow‐up) for the control group (CON, pink line, circles) and the intervention group (INT, blue line, triangles). Thin lines represent individual cognitive trajectories; small circles, triangles, and the shaded areas represent the distribution of individual participant scores per group and timepoint. (B) Standardized change scores in EF at t1 and t2 for the CON and INT. (C) Transitions of participants between distribution‐based clinically meaningful improvement (CMI), robust CMI (rCMI), or no CMI in EF from t1 to t2 for the CON and INT. (D) Estimated marginal means of domain‐wise secondary cognitive outcomes over time for the INT and CON. (E) Transition of participants meeting criteria for mild cognitive impairment (MCI) over time in the CON and INT. EF, executive functions; (r)CMI, (robust) clinically meaningful improvement; CON, control group; INT, intervention group; MCI, mild cognitive impairment; SD, standard deviation; t0, baseline; t1, 6‐weeks post‐allocation; t2, 6‐month follow‐up; WM, working memory.

Descriptive data and results of LME models for primary and secondary outcomes are reported in Table 2 and Table S2. For the executive functions composite score (Figure 2A), the primary outcome, the LME model revealed a significant time*group interaction (*F*(2,148.978) = 7.034, *p *= 0.0012, *P*_FDR _= 0.0018), with a medium effect size at t1 (Cohen's *d *= 0.500, 95% CI: 0.221–0.779) and a small effect size at t2 (Cohen's *d *= 0.397, 95% CI: 0.115–0.679). Although the performance of INT increased over time (Delta*_t_*_1–_ *_t_*_0 _= 0.342, standard error [SE] = 0.050; Delta*_t_*_2–_ *_t_*_0 _= 0.294, SE = 0.051), the performance of the CON group remained relatively stable (Delta*_t_*_1–_ *_t_*_0 _= 0.094, SE = 0.049; Delta*_t_*_2–_ *_t_*_0 _= 0.097, SE = 0.049). Exploratory analyses indicate that participants in the INT were more likely to show an rCMI in executive functions than those in the CON at both t1 (OR = 4.038, 95% CI: 1.248–15.767) and t2 (OR = 3.365, 95% CI: 1.004–13.381). At t1, 31.6% (12/38) of participants in the INT exhibited a rCMI compared to 10.3% (4/39) in the CON, and at t2, 27.8% (10/36) vs 10.3% (4/39), respectively (Figure 2B,C).

**TABLE 2:** Results of LME models to assess training effects in primary and secondary outcomes.

				Absolute change score based on EMM (SE)				timepoint × group	
	ANOVA of LME models			Control group (CON)		Intervention group (INT)		Cohen's d (95% Confidence Interval)	
	Timepoint	Group	Timepoint × Group	t1–t0	t2–t0	t1–t0	t2–t0	t1–t0	t2–t0
Executive functions	F(2,148.978) = 23.536,	F(1,80.011) = 1.033,	F(2,148.978) = 7.034,	0.094	0.097	0.342	0.342	0.500	0.397
	*P* = ≤ 0.0001, *P*_FDR_ = ≤ 0.0001	*P* = 0.3126, *P*_FDR_ = 0.3126	*P* = 0.0012, *P*_FDR_ = 0.0018	(0.049)	(0.049)	(0.05)	(0.05)	(0.221–0.779)	(0.115–0.679)
Attention & working memory	F(2,148.888) = 3.577,	F(1,79.950) = 0.348,	F(2,148.888) = 1.291,	0.036	0.045	0.165	0.165	0.225	0.157
	*P* = 0.0304, *P*_FDR_ = 0.0760	*P* = 0.5568, *P*_FDR_ = 0.6960	*P* = 0.2781, *P*_FDR_ = 0.5771	(0.058)	(0.058)	(0.059)	(0.059)	(‐0.060–0.509)	(‐0.130–0.445)
Memory	F(2,148.667) = 17.212,	F(1,79.697) = 0.153,	F(2,148.667) = 0.456,	0.117	0.283	0.182	0.182	0.103	−0.033
	*P* = ≤ 0.0001, *P*_FDR_ = ≤ 0.0001	*P* = 0.6972, *P*_FDR_ = 0.6972	*P* = 0.6345, *P*_FDR_ = 0.6972	(0.065)	(0.065)	(0.066)	(0.066)	(‐0.186–0.392)	(‐0.325–0.258)
Visuo‐cognition	F(2,147.964) = 7.271,	F(1,79.047) = 0.682,	F(2,147.964) = 0.783,	0.037	0.078	0.077	0.077	0.096	0.183
	*P* = 0.0010, *P*_FDR_ = 0.0049	*P* = 0.4116, *P*_FDR_ = 0.6173	*P* = 0.4588, *P*_FDR_ = 0.6256	(0.042)	(0.042)	(0.043)	(0.043)	(‐0.191–0.384)	(‐0.107–0.474)
Language	F(2,148.203) = 4.222,	F(1,78.411) = 1.054,	F(2,148.203) = 0.402,	0.086	0.025	0.117	0.117	0.083	0.173
	*P* = 0.0165, *P*_FDR_ = 0.0618	*P* = 0.3078, *P*_FDR_ = 0.5771	*P* = 0.6699, *P*_FDR_ = 0.6972	(0.049)	(0.049)	(0.05)	(0.05)	(‐0.295–0.462)	(‐0.209–0.556)
Global cognition	F(2,148.159) = 33.715,	F(1,79.682) = 0.865,	F(2,148.159) = 3.903,	0.073	0.105	0.173	0.173	0.285	0.224
	*P* = ≤ 0.0001, *P*_FDR_ = ≤ 0.0001	*P* = 0.3552, *P*_FDR_ = 0.5920	*P* = 0.0223, *P*_FDR_ = 0.0668	(0.027)	(0.027)	(0.027)	(0.027)	(0.072–0.498)	(0.009–0.439)
Multi‐SubCoDE SCD‐Severity	F(2,139.455) = 7.607,	F(1,77.597) = 1.046,	F(2,139.455) = 0.528,	−1.303	−0.27	−1.236	−1.236	0.011	−0.096
	*P* = 0.0007, *P*_FDR_ = 0.0088	*P* = 0.3097, *P*_FDR_ = 0.4645	*P* = 0.5909, *P*_FDR_ = 0.6446	(0.469)	(0.473)	(0.453)	(0.453)	(‐0.207–0.229)	(‐0.317–0.126)
BDI‐II total score	F(2,140.395) = 3.958,	F(1,78.433) = 2.377,	F(2,140.395) = 1.656,	−1.307	−1.403	−0.182	−0.182	0.187	0.168
	*P* = 0.0213, *P*_FDR_ = 0.0850	*P* = 0.1271, *P*_FDR_ = 0.3814	*P* = 0.1946, *P*_FDR_ = 0.4425	(0.491)	(0.495)	(0.475)	(0.475)	(‐0.037–0.412)	(‐0.060–0.396)
FSMCF total score	F(2,140.097) = 4.449,	F(1,78.114) = 1.441,	F(2,140.097) = 0.072,	−2.66	−0.929	−2.653	−2.653	0.000	−0.038
	*P* = 0.0134, *P*_FDR_ = 0.0804	*P* = 0.2337, *P*_FDR_ = 0.4425	*P* = 0.9305, *P*_FDR_ = 0.9305	(1.281)	(1.292)	(1.239)	(1.239)	(‐0.221–0.222)	(‐0.263–0.187)
SF‐36 total score	F(2,142.307) = 0.562,	F(1,78.227) = 1.297,	F(2,142.307) = = 0.961,	−0.328	0.043	1.674	1.674	0.118	−0.074
	*P* = 0.5716, *P*_FDR_ = 0.6446	*P* = = 0.2582, *P*_FDR_ = 0.4425	*P* = 0.3848, *P*_FDR_ = 0.5131	(1.638)	(1.654)	(1.607)	(1.607)	(‐0.150–0.386)	(‐0.346–0.198)
MDS‐UPDRS‐III total score	F(2,147.827) = 0.157,	F(1,80.382) = 1.413,	F(2,147.827) = 0.152,	0.434	−0.169	0.067	0.088	−0.079	0.055
	*P* = 0.8550, *P*_FDR_ = 0.8593	*P* = 0.2381, *P*_FDR_ = 0.8593	*P* = 0.8593, *P*_FDR_ = 0.8593	(0.771)	(0.778)	(0.778)	(0.808)	(‐0.545–0.387)	(‐0.422–0.533)
Purdue Pegboard both hands	F(2,146.205) = 0.455,	F(1,77.868) = 0.324,	F(2,146.205) = 0.918,	−0.15	−0.123	0.068	0.068	0.189	0.080
	*P* = 0.6354, *P*_FDR_ = 0.8593	*P* = 0.5707, *P*_FDR_ = 0.8593	*P* = 0.4018, *P*_FDR_ = 0.8593	(0.112)	(0.112)	(0.115)	(0.115)	(‐0.088–0.466)	(‐0.200–0.359)
Purdue Pegboard assembly	F(2,148.408) = 0.359,	F(1,79.717) = 0.034,	F(2,148.408) = 3.237,	−0.17	−0.127	0.21	0.21	0.390	0.413
	*P* = 0.6989, *P*_FDR_ = 0.8593	*P* = 0.8539, *P*_FDR_ = 0.8593	*P* = 0.0421, *P*_FDR_ = 0.3787	(0.124)	(0.124)	(0.127)	(0.127)	(0.030–0.751)	(0.049–0.777)

Abbreviations: ANOVA, analysis of variance; BDI‐II, Beck Depression Inventory; EMM, estimated marginal means; FSMCF, Fatigue Scale for Motor and Cognitive Functions; LME, linear mixed effects; MDS‐UPDRS‐III, Movement Disorder Society Unified Parkinson's Disease Rating Scale Part 3; Multi‐SubCoDE, Multiple Domain Subjective Cognitive Decline Evaluation; SE, standard error; SF‐36, Short‐Form 36.

Despite a similar trend for global cognition, the training effects missed the FDR‐corrected significance threshold (*F*(2,148.159) = 3.903, *p *= 0.0223, *P*_FDR _= 0.0668). No significant time*group interactions were observed for other domain‐wise secondary cognitive outcomes (Figure 2D).

Exploratory analyses of individual cognitive test scores (Tables S3 and S4) revealed that observed cognitive training effects were primarily driven by positive training‐related improvements in logical reasoning (Leistungsprüfsystem für 50‐ bis 90‐Jährige, LPS‐50+ Subtest 4), working memory (Digit Span Backward), set‐shifting (Trail Making Test B/A), selective attention (Brief Test of Attention), and short‐term memory (Digit Span Forward). Despite a descriptive tendency for a positive training effect on MCI status, the time*group interaction was not significant (Figure 2E).

For executive functions, global cognition, memory, visuo‐cognition, and MCI status, significant main effects of timepoint were found, indicating general improvements in test performance and a decreasing likelihood of meeting MCI criteria over time, independent of group assignment.

No significant time*group interactions were observed for secondary motor outcomes (MDS‐UPDRS‐III, Purdue Pegboard both‐hands, Purdue Pegboard assembly) and secondary PROMs (Multi‐SubCoDE SCD‐Severity, BDI‐II total score, FSMCF total score, SF‐36 total score). However, for PROMs including subjective cognitive decline (Multi‐SubCoDE SCD‐Severity, *F*(2,139.455) = 7.607, *p* ≤ 0.0001, *P*_FDR _= 0.0088), depressive symptoms (BDI‐II total score, *F*(2,140.395) = 3.958, *p *= 0.0213, *P*_FDR _= 0.0850), and fatigue (FSMCF total score, *F*(2,140.097) = 4.449, *p *= 0.0134, *P*_FDR _= 0.0804), main effects of timepoint were found, indicating reduced symptom severity over time independent of group assignment.

### 3.3 Exploratory prediction of training responsiveness

**FIGURE 3:**
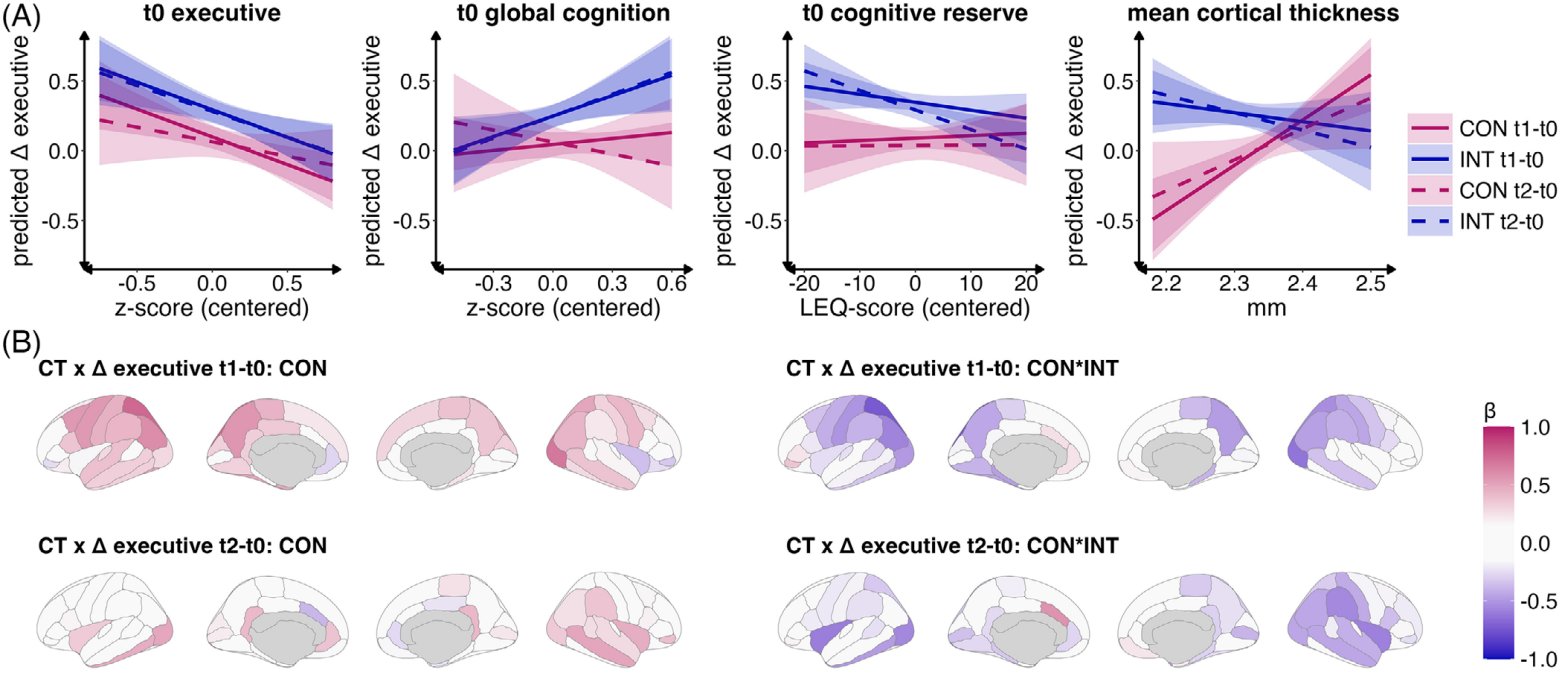
Exploratory prediction of cognitive training responsiveness. (A) Predicted change (Δ) in executive functions as a function of baseline (t0) executive performance, t0 global cognition, cognitive reserve (operationalized by the Lifetime of Experiences Questionnaire [LEQ], score of young adulthood), and mean cortical thickness. Lines show group‐wise (CON, control group, pink; INT, intervention group, blue) model‐based predictions per timepoint (Δt1–t0, solid lines; Δt2–t0, dashed lines). Shaded areas represent 95% confidence intervals. Predictions are based on regression models evaluated across the observed range of mean cortical thickness. (B) ROI‐wise standardized β regression coefficients of the executive change scores (Δ executive) of the CON and the *group interaction term to predict cortical thickness controlled for t0 executive performance, age, and cognitive reserve. Separate models were calculated per timepoint (t1–t0 and t2–t0). CON, control group; CT, cortical thickness; INT, intervention group; LEQ, Lifetime of Experiences Questionnaire; t0, baseline; ROI, region of interest.

Exploratory analyses on the prediction of training responsiveness with multiple multivariable linear regression models revealed that higher intervention responsiveness in executive functions at both t1 and t2 was associated with lower baseline executive performance, higher global cognition, and lower cognitive reserve (LEQ,^49^ in young adulthood). Whereas higher mean cortical thickness was associated with greater retest improvement in the CON, the reversed association was observed in the INT (Figure 3A). Further whole‐brain region of interest (ROI) analyses revealed parieto‐occipital ROIs to primarily drive this difference: higher mean cortical thickness in the right lateral occipital gyrus and left inferior and superior parietal gyrus was associated with greater retest improvement in the CON. The group interaction was significantly negative, indicating no such associations or even reverted associations for these areas in the INT at FDR‐corrected significance threshold (Figure 3B).

## 4 DISCUSSION

In the parallel‐group phase of the CogTrAiL‐RBD study, we evaluated the feasibility and efficacy of a 5‐week, digital, adaptive, multidomain cognitive training within a lifestyle‐guidance framework in individuals with iRBD, indicating an early α‐synucleinopathy and possible prodromal phase of PD with or without dementia. Our main findings were: (1) the intervention was highly feasible; (2) the INT group showed positive effects on the primary outcome of executive function compared to the CON both 6 weeks and 6 months post‐allocation, with moderate (t1) and small (t2) effect sizes; and (3) intervention effects on secondary cognitive and motor outcomes, as well as PROMs, were non‐significant. Exploratory analyses indicated that 4) greater intervention responsiveness in the primary outcome was associated with lower baseline executive performance, higher baseline global cognition, lower cognitive reserve, and lower baseline mean cortical thickness.

Our findings demonstrate that participation in a short, digital, adaptive, multidomain cognitive training combined with a healthy lifestyle module is both feasible and capable of producing clinically meaningful improvements in executive functions in individuals with iRBD. The sustained effects at 6 months, albeit smaller than immediately post‐intervention, suggest that even relatively brief cognitive interventions may lead to lasting cognitive benefits in this at‐risk population and are in line with meta‐analytically reported effect sizes following computerized cognitive training in PD.^18^, ^19^, ^20^ Nevertheless, the trial's design may conflate several potentially active ingredients (as further discussed below) and only allowed for the assessment of short‐term cognitive benefits. The intervention's potential for scalable secondary dementia prevention and disease modification requires evaluation in larger and longer trials with clinical progression and/or conversion endpoints.^28^

As expected from the cognitive training literature,^11^, ^12^, ^20^ effects were even smaller for global cognition, and despite descriptive trends, we did not observe statistically significant intervention effects in other cognitive domains. The latter may be partly be explained by the fact that cognitive training effects are generally expected to be larger in domains known to be particularly vulnerable in PD.^18^, ^19^, ^20^ In addition, higher difficulty levels in the multidomain cognitive training were characterized by increased task complexity and a strong executive component. This design may explain the observed pattern of stronger near‐transfer effects in executive functioning and weaker far‐transfer effects in other cognitive domains, a common finding in targeted, single‐domain cognitive training approaches such as working memory training.^57^

Exploratory analyses of individual cognitive test scores indicated that the observed cognitive training effects were primarily driven by improvements in LPS‐50+ Subtest 4, Digit Span Forward and Backward, the Trail Making Test B/A, and the Brief Test of Attention. Of note, these neuropsychological task paradigms were not directly trained within HeadApp/NEUROvitalis Digital. Rather, they rely on cognitive processes specifically targeted by the training (e.g., set‐shifting, interference control, planning, working memory), and therefore likely reflect near‐transfer effects rather than direct, task‐specific “train‐the‐task” effects. This is further supported by a lack of training effects in the language domain as well as both verbal fluency measures, despite well‐described language alterations in LBD.^58^ This pattern reinforces the notion that observed gains were not driven by global cognitive enhancement or semantic system modulation but instead reflect selective improvements in executive‐attentional processes.

We found improved cognitive performance over time irrespective of group assignment for several cognitive domain composite scores. This pattern may reflect practice effects that were not fully mitigated by the use of parallel test forms. Although typically considered a source of bias in repeated cognitive assessments, practice effects are also recognized as a prognostic marker of cognitive performance and dementia risk in aging populations,^59^, ^60^ which may even be used for stratification in clinical trials.^61^ Future work should explicitly evaluate the role of practice effects in cognitive diagnostics in iRBD. As discussed in the Alzheimer's field, so‐called “run‐in” periods with repeated cognitive assessments prior to randomization may substantially improve the efficiency of clinical trials by yielding clearer estimates of decline and thus smaller required sample sizes.^61^

As outlined previously, iRBD may represent a particularly well‐defined window for implementing neuroprotective and cognition‐preserving interventions. iRBD is associated with an exceptionally high risk of future cognitive and motor decline while simultaneously offering a relatively long and clinically identifiable prodromal phase. Together, the potential for heightened neuroplasticity in this prodromal state and the opportunity to meaningfully attenuate neurodegenerative processes before phenoconversion make iRBD a highly promising target population for early interventions.^62^ Given the lack of established disease‐modifying pharmacological treatments, home‐based, low‐risk, and easily accessible lifestyle interventions appear particularly well‐suited for this group. Indicated by a high participation rate, low study dropouts, and high protocol adherence, the CogTrAiL‐RBD trial demonstrated high feasibility. The digital cognitive training featured an age‐appropriate, user‐friendly interface, with daily variation in exercises and difficulty levels adapting to individual performance, avoiding under‐challenge or overstrain. Furthermore, a consistent contact person, accessibility of this contact person by phone or email, welcome and/or closing of study visits with the contact person being present, written prior information on all study procedures, calendar reminders, and seamless integration of the trial within the local clinical and research network may have contributed to the overall high feasibility.^63^ Drawing on experience from the CogTrAiL‐RBD trial and informal participant feedback, it is conceivable that combining pharmacological and non‐pharmacological interventions could enhance participation rates, adherence, and motivation in future pharmacological trials.

We did not observe relevant intervention effects on secondary motor outcomes or PROMs. However, significant main effects of time indicated reduced symptom severity in subjective cognitive decline, depressive symptoms, and fatigue, regardless of group assignment. This pattern suggests that simply participating in the CogTrAiL‐RBD study itself may have positively influenced participants’ well‐being and self‐perceived symptom burden, indicating that study enrollment alone may have conferred a meaningful psychological benefit. This finding should be interpreted with caution as it may reflect study site–specific factors discussed previously in the context of feasibility.

Given the heterogeneity of intervention effects across participants, we conducted exploratory follow‐up analyses to identify predictors of training responsiveness.^64^ Consistent with both the compensation and magnification accounts of cognitive plasticity^65^, ^66^ and similar to findings in PD,^67^ our results suggest that both room for improvement and existing cognitive resources are important for responsiveness to cognitive interventions. Notably, the strength and/or specificity of these associations in the INT indicate that they reflect true training‐related associations rather than regression to the mean or predictors of retest/practice effects.^68^ In addition, in the INT, lower cognitive reserve and lower mean cortical thickness were associated with greater cognitive intervention responsiveness, whereas these associations were attenuated or reversed in the CON. We hypothesize that the CogTrAiL‐RBD intervention may have served as a booster for cognitive reserve, thereby reducing the reliance on structural brain reserve for cognitive plasticity in individuals with iRBD.^69^, ^70^, ^71^ Further enrichment markers, such as cognitive status, as well as clinical characteristics including subtle motor symptoms and olfactory function, contributing to the clinical heterogeneity in iRBD, may have additionally influenced individual intervention responsiveness, and warrant further investigation in larger samples.

The main limitation of the study is the absence of an engagement‐matched active control group. Consequently, it is not possible to determine whether the observed effects reflect specific or non‐specific intervention effects. The combined intervention also conflates several potentially active components (digital cognitive training, psychoeducational content, activity self‐monitoring, and general engagement), precluding attribution of effects to individual components. Of note, personal contact was largely comparable between groups; however, it was not systematically tracked. During the intervention period, personal contact was only established in case of problems with either intervention component.

Moreover, the healthy lifestyle module was limited to psychoeducation and digital activity diaries, which differs substantially from large‐scale multidomain prevention trials in other at‐risk cohorts.^2^, ^4^, ^5^, ^6^, ^7^, ^8^ Effect sizes observed in long‐term multidomain lifestyle interventions are not necessarily the sum of their individual components^3^ and may, in some cases, be comparable to those of short‐term digital cognitive training alone.^9^, ^10^, ^20^ However, it is plausible that longer and more intensive interventions, such as face‐to‐face, personalized counseling, or multimodal intervention packages addressing lifestyle domains beyond cognitive training (e.g., physical activity, nutritional counseling, or psychotherapy), could yield more sustained effects and broader benefits across different health domains, thereby contributing to overall brain and cognitive health. Accordingly, effects observed in short‐term cognitive training interventions versus long‐term multidomain lifestyle interventions likely reflect different underlying mechanisms and intervention targets.

Another limitation of the present study is the modest sample size. The study was underpowered to reliably detect (small) effects in secondary and exploratory outcomes. Nevertheless, the longitudinal, delayed‐start design may provide valuable insights into the behavioral and neurobiological mechanisms underlying cognitive intervention responsiveness with future analyses.

Taken together, our findings suggest that early, low‐threshold cognitive interventions may primarily strengthen executive functioning in individuals with iRBD at risk for cognitive decline and a clinical progression toward PD with or without dementia, with responsiveness depending on both cognitive vulnerability and resources.

## AUTHOR CONTRIBUTIONS

**Anja Ophey**: Conceptualization, Methodology, Software, Formal analysis, Investigation, Resources, Data Curation, Writing – Original Draft, Visualization, Project Administration, Funding Acquisition, and Supervision. **Sinah Röttgen**: Validation, Formal analysis, Investigation, Data Curation, Writing – Review & Editing, and Project Administration. **Konstantin Kufer**: Validation, Formal analysis, Investigation, Data Curation, and Writing – Review & Editing. **Julia Pauquet**: Software, Investigation, and Writing – Review & Editing. **Daniel Scharfenberg**: Software, Investigation, and Writing – Review & Editing. **Philipp Armbrust**: Investigation, Data Curation, and Writing – Review & Editing. **Anastasia Kammerzell**: Investigation, Data Curation, and Writing – Review & Editing. **Nathalie Knopf**: Investigation, Data Curation, and Writing – Review & Editing. **Sandy Kollath**: Investigation and Writing – Review & Editing. **Philipp Sommer**: Investigation, Data Curation, and Writing – Review & Editing. **Christopher E. J. Doppler**: Investigation and Writing – Review & Editing. **Ezequiel Farrher**: Software and Writing – Review & Editing. **Aline Seger**: Investigation and Writing – Review & Editing. **N. Jon Shah**: Resources and Writing – Review & Editing. **David Zopfs**: Resources and Writing – Review & Editing. **Kathrin Reetz**: Resources and Writing – Review & Editing. **Gereon R. Fink**: Conceptualization, Methodology, Resources, and Writing – Review & Editing. **Michael Sommerauer**: Conceptualization, Methodology, Software, Validation, Resources, Writing – Review & Editing, and Supervision. **Elke Kalbe**: Conceptualization, Methodology, Resources, Writing – Review & Editing, and Supervision.

## CONFLICT OF INTEREST STATEMENT

Sinah Röttgen, Konstantin Kufer, Julia Pauquet, Daniel Scharfenberg, Nathalie Knopf, Sandy Kollath, Philipp Sommer, Christopher E. J. Doppler, Ezequiel Farrher, Aline Seger, N. Jon Shah, David Zopfs: None. Anja Ophey received funding from the Koeln Fortune Program/Faculty of Medicine, University of Cologne, the Novartis Stiftung für therapeutische Forschung, the Imhoff foundation, Prof. Klaus Thiemann Stiftung, the International Conference on Alzheimer's and Parkinson's Diseases (AD/PD), and the German Academic Exchange Service (DAAD). Philipp Armbrust received funding from the Koeln Fortune Program/Faculty of Medicine, University of Cologne. Anastasia Kammerzell received funding from the Koeln Fortune Program/Faculty of Medicine, University of Cologne. Kathrin Reetz received grants from the Enroll (Cure Huntington's Disease Initiative, CHDI) registry, Roche clinical trial, NovoNordisk clinical trial, Eisai registry, Lilly registry, and Friedreich's Ataxia Research Alliance (FARA) registry; received consulting fees from Lilly, Eisai, and Novartis; received lecture honoraria from Lilly, Eisai, and Biogen; participates in the Data Safety Monitoring BoardDSMB for a clinical trial, and the scientific advisory board of the European Huntington's Disease Network (EHDN) and FARA; and is president of the German Brain Foundation. Gereon R. Fink received grants from the Deutsche Forschungsgemeinschaft (DFG, German Research Foundation); receives royalties from Springer, Schattauer, Thieme, Elsevier, and Hogrefe; received lecture honoraria from Forum für Medizinische Fortbildung (FoMF) and Deutsche Gesellschaft für Neurologe (DGN), and is board member of the European Academy of Neurology. Michael Sommerauer received funding from the Federal Ministry of Research, Technology and Space (BMFTR), the European Union (EU) Horizon Europe Research and Innovation Programme, and the European Research Council; received honoraria from Bial and Desitin. Elke Kalbe is author of the cognitive training program NEUROvitalis (Prolog/ HeadApp GmbH, Germany), but does not receive honoraria in this context. Author disclosures are available in the Supporting Information.

## CONSENT STATEMENT

Written informed consent to participate in the CogTrAiL‐RBD study was obtained from all participants.

## TRIAL REGISTRATION

German Clinical Trials Register (DRKS00024898, https://drks.de/search/de/trial/DRKS00024898).

## Supporting information




Supporting Information

## Data Availability

The data underlying this study, together with a corresponding data dictionary, are available from the corresponding author upon reasonable request. Data are not publicly available because of privacy and ethical restrictions. In particular, full anonymization is not currently feasible. Access to de‐identified data may be granted to qualified researchers for scientific purposes, subject to review and approval by the study investigators and in accordance with applicable data protection regulations and institutional policies.
