# Supplementary material for: Cognitive training and promoting a healthy lifestyle in isolated REM sleep behavior disorder: The randomized controlled trial CogTrAiL‐RBD
Source: Alzheimers Dement. 2026 Aug 5;22(8):e71707. doi: 10.1002/alz.71707 (PMC13440496; doi:10.1002/alz.71707)
Supplement: Supplementary file 1 [file ALZ-22-e71707-s001.pdf]

SUPPLEMENTARY MATERIAL

Cognitive training and promoting a healthy lifestyle in isolated REM sleep behavior disorder: The randomized controlled trial CogTrAiL-RBD

Ophey*, Röttgen* et al.

Supplementary Material 1. Details on the Magnetic Resonance Imaging (MRI) Protocol and Preprocessing -- p. 2

Supplementary Material 2. Details on the Definition of Robust and Distribution-based Clinically Meaningful Improvement -- p. 3

Supplementary Table 1. Group-wise Medication Profiles at Baseline-- p. 4

Supplementary Table 2. Group-wise Descriptive Data Per Timepoint for Primary and Secondary Outcomes --- p. 5

Supplementary Table 3. Group-wise Descriptive Data Per Timepoint for Exploratory Outcomes -- p. 6

Supplementary Table 4. Results of LME Models to Assess Training Effects in Exploratory Outcomes -- p. 7

Supplementary Material 1. Details on the Magnetic Resonance Imaging (MRI) Protocol and Preprocessing

Brain imaging was conducted at the Research Center Juelich with a 3T SIEMENS PRISMA (Siemens Healthineers, Erlangen, Germany) scanner equipped with a 64-channel receive head coil. T1-weighted brain images were acquired using a magnetization-prepared rapid acquisition gradient echo (MPRAGE) sequence (2500 ms repetition time, 2.22 ms echo time, 7 degree flip angle, 195.5×241×241 mm^3 field of view, 208×256×256 matrix size, 0.94×0.94×0.94 mm^3 voxel size).

Structural T1-weighted images were processed using FreeSurfer's automated cortical reconstruction pipeline (version 7.4.1, http://surfer.nmr.mgh.harvard.edu). The workflow included intensity non-uniformity correction, skull stripping, white-gray matter boundary segmentation, and reconstruction of the white and pial surfaces with topology correction. To further reduce random noise (e.g., scanner fluctuations), we applied the FreeSurfer longitudinal pipeline to each participant, using all available MRI data across all timepoints. Trained analysts visually inspected segmentation outputs, and all scans met quality-control criteria. Cortical regions were parcellated using the Desikan-Killiany atlas. Mean cortical thickness values were extracted from the resulting parcellation.

Supplementary Material 2. Details on the Definition of Robust and Distribution-based Clinically Meaningful Improvement

In addition to reporting the analyses based on the raw executive composite score, we report robust and distribution-based clinically meaningful improvement (robust CMI, rCMI). CMI was defined as an improvement of ≥ 0.5 standard deviations (SD) from t0 to each follow-up timepoint (t1 or t2) in the weighted composite score of executive functions. For each participant, we calculated the standardized change score (SCS) by dividing the absolute change scores ($Delta_{t1-t0}$ and $Delta_{t2-t0}$) by the CON's t0 SD. To evaluate whether a CMI was also statistically reliable, we calculated the SCS threshold for reliable improvement at one-sided $\alpha = 0.05$ using the CON's standard error of the difference (SEdiff), which accounts for expected measurement variability between repeated assessments. Participants exceeding both the CMI and reliability thresholds were classified as showing rCMI.

Supplementary Table 1. Group-wise Medication Profiles at Baseline

	Control Group<br>*n* = 36*	Intervention Group<br>*n* = 36*
RBD-specific: melatonin	3 (8.33%)	3 (8.33%)
RBD-specific: clonazepam	1 (2.78%)	1 (2.78%)
RBD-specific: zopiclone	0 (0.00%)	1 (2.78%)
Supplements and alternative medicine	12 (33.33%)	7 (19.44%)
Antidepressants	1 (2.78%)	3 (8.33%)
Mood stabilizers	1 (2.78%)	1 (2.78%)
Antihyperintensives	11 (30.56%)	19 (52.78%)
Antiplatelets and anticoagulants	7 (19.44%)	10 (27.78%)
Lipid-lowering agents	10 (27.78%)	12 (33.33%)
Endocrine and metabolic	8 (22.22%)	9 (25.00%)
Respiratory	4 (11.11%)	3 (8.33%)
Gastrointestinal	5 (13.89%)	7 (19.44%)
Urological	5 (13.89%)	4 (11.11%)
Pain and inflammation	2 (5.56%)	2 (5.56%)

Data are *n* (%). RBD-specific: melatonin, clonazepam or zopiclone; Supplements and alternative medicine: vitamins, minerals, and homeopathic remedies; Antidepressants: Serotonin reuptake inhibitors and related agents; Mood stabilizers: Anticonvulsants and antipsychotics used for mood regulation; Antihypertensives: Agents for lowering blood pressure; Antiplatelets and anticoagulants: antithrombotic medication; Lipid-lowering agents: Drugs for reducing cholesterol or lipids; Endocrine and metabolic: Hormones and agents for metabolic disorders; Respiratory: Medications for lung or airway conditions; Gastrointestinal: Drugs for stomach, intestine, or digestive disorders; Urological: Medications for urinary or prostate conditions; Pain and inflammation: Analgesics and anti-inflammatory agents.

*Medication data was not available for *n* = 6 in the CON, and *n* = 4 in the INT.

Supplementary Table 2. Group-wise Descriptive Data Per Timepoint for Primary and Secondary Outcomes

	Control Group			Intervention Group		
	**t0**	**t1**	**t2**	**t0**	**t1**	**t2**
	n = 42	*n* = 39	*n* = 39	*n* = 40	*n* = 38	*n* = 36
Executive Functions	0.368<br>(0.463)<br>[-0.307 - 1.419]	0.458<br>(0.491)<br>[-0.468 - 1.565]	0.460<br>(0.488)<br>[-0.504 - 1.559]	0.326<br>(0.535)<br>[-1.188 - 1.199]	0.676<br>(0.523)<br>[-0.553 - 1.493]	0.650<br>(0.523)<br>[-0.824 - 1.593]
Attention & Working Memory	0.494<br>(0.595)<br>[-0.595 - 1.992]	0.559<br>(0.629)<br>[-0.758 - 1.752]	0.545<br>(0.575)<br>[-0.741 - 2.010]	0.495<br>(0.559)<br>[-0.567 - 1.490]	0.675<br>(0.665)<br>[-0.841 - 2.318]	0.654<br>(0.594)<br>[-0.394 - 2.303]
Memory	0.231<br>(0.626)<br>[-1.109 - 1.388]	0.345<br>(0.712)<br>[-0.983 - 1.683]	0.513<br>(0.633)<br>[-0.826 - 1.716]	0.270<br>(0.643)<br>[-1.208 - 1.683]	0.487<br>(0.733)<br>[-0.926 - 1.780]	0.562<br>(0.616)<br>[-1.014 - 1.722]
Visuo-Cognition	0.503<br>(0.365)<br>[-0.383 - 1.273]	0.538<br>(0.424)<br>[-0.258 - 1.248]	0.575<br>(0.461)<br>[-0.736 - 1.548]	0.540<br>(0.467)<br>[-0.552 - 1.373]	0.638<br>(0.501)<br>[-0.549 - 1.648]	0.719<br>(0.400)<br>[-0.460 - 1.360]
Language	0.220<br>(-0.080, 0.375)<br>[-0.825 - 1.055]	0.325<br>(0.065, 0.530)<br>[-0.660 - 1.055]	0.220<br>(-0.095, 0.430)<br>[-0.645 - 1.155]	0.325<br>(-0.143, 0.530)<br>[-0.735 - 0.845]	0.383<br>(0.070, 0.605)<br>[-0.735 - 1.015]	0.350<br>(0.065, 0.545)<br>[-0.660 - 1.000]
Global Cognition	0.348<br>(0.337)<br>[-0.245 - 1.229]	0.429<br>(0.391)<br>[-0.411 - 1.255]	0.454<br>(0.354)<br>[-0.211 - 1.160]	0.364<br>(0.372)<br>[-0.544 - 0.931]	0.562<br>(0.439)<br>[-0.652 - 1.236]	0.575<br>(0.377)<br>[-0.349 - 1.228]
Multi-SubCoDE SCD-Severity*[a]	5<br>(2, 9)<br>[0 - 23]	4<br>(1, 7)<br>[0 - 19]	5<br>(2, 9)<br>[0 - 26]	6<br>(4, 12)<br>[0 - 24]	5.500<br>(2, 9)<br>[0 - 26]	5.500<br>(2, 10)<br>[0 - 30]
BDI-II*[a]	5<br>(1, 9)<br>[0 - 17]	2<br>(1, 6)<br>[0 - 17]	2.500<br>(1, 7)<br>[0 - 16]	4<br>(1, 9)<br>[0 - 28]	3<br>(1, 8)<br>[0 - 28]	3.500<br>(2, 7.500)<br>[0 - 29]
FSMCF*[a]	35<br>(23, 47)<br>[20 - 70]	30<br>(22, 40)<br>[20 - 67]	31.500<br>(22, 46)<br>[20 - 75]	37<br>(25, 50)<br>[20 - 88]	30.500<br>(24, 48)<br>[20 - 79]	33.500<br>(23, 51)<br>[20 - 84]
SF-36 total*[a]	83.938<br>(72.979, 93)<br>[40.938 - 99.625]	84.625<br>(71.698, 92.438)<br>[32.563 - 100]	84.813<br>(75.188, 91.219)<br>[36.375-99.625]	83.188<br>(66.313, 89.625)<br>[21.750-97.250]	87<br>(69.938, 92.875)<br>[27.438-97.375]	83.406<br>(71.188, 89.688)<br>[23.813-98.375]
MDS-UPDRS-III	5<br>(3, 8)<br>[0 - 19]	6<br>(2, 8)<br>[1 - 19]	4<br>(2, 7)<br>[0 - 15]	5.500<br>(2.500, 9)<br>[0 - 23]	5.500<br>(3, 10)<br>[0 - 19]	5<br>(3, 8)<br>[0 - 21]
Purdue Pegboard Both Hands*[b]	-0.600<br>(-1.300, 0.070)<br>[-2.940 - 4.000]	-0.840<br>(-1.530, 0.070)<br>[-2.940 - 2.070]	-0.840<br>(-1.300, -0.160)<br>[-3.460 - 2.620]	-0.390<br>(-0.840, 0.070)<br>[-2.900 - 1.100]	-0.390<br>(-1.100, 0.140)<br>[-2.150 - 0.750]	-0.490<br>(-1.090, 0.010)<br>[-2.420 - 0.750]
Purdue Pegboard Assembly*[b]	-0.044<br>(0.961)<br>[-2.240 - 2.390]	-0.201<br>(1.195)<br>[-2.440 - 2.950]	-0.210<br>(1.141)<br>[-2.410 - 2.370]	-0.340<br>(0.978)<br>[-2.170 - 1.840]	-0.102<br>(0.947)<br>[-2.430 - 1.380]	-0.076<br>(0.905)<br>[-1.810 - 1.650]

Notes. Data are mean (standard deviation) [range: minimum - maximum] or median (inter quartile range) [range: minimum - maximum]. BDI-II, Beck Depression Inventory; FSMCF, Fatigue Scale for Motor and Cognitive Functions; MDS-UPDRS-III, Movement Disorder Society Unified Parkinson’s Disease Rating Scale; SF-36, Short-Form 36.
* Data not available for all randomized participants.
[a] missing for *n* = 3 / 4 / 3 in the CON at t0 / t1 / t2 respectively, and *n* = 1 / 0 / 0 in the INT at t0 / t1 / t2 respectively
[b] missing for *n* = 1 / 0 / 0 in the INT at t0 / t1 / t2 respectively

Supplementary Table 3. Group-wise Descriptive Data Per Timepoint for Exploratory Outcomes

	Control Group			Intervention Group		
	**t0**<br>*n* = 42	**t1**<br>*n* = 39	**t2**<br>*n* = 393	**t0**<br>*n* = 40	**t1**<br>*n* = 38	**t2**<br>*n* = 36
LPS50+ Subtest 4 Logical Reasoning	0.288<br>(0.778)<br>[-1.500 - 2.100]	0.331<br>(0.583)<br>[-0.600 - 1.500]	0.426<br>(0.724)<br>[-1.200 - 2.200]	0.075<br>(0.783)<br>[-1.500 - 2.000]	0.643<br>(0.767)<br>[-1.300 - 2.150]	0.494<br>(0.693)<br>[-1.100 - 2.000]
Stroop Interference	0.850<br>(0.500, 1.200)<br>[-0.300 - 1.900]	1.100<br>(0.800, 1.400)<br>[-0.100 - 2.200]	1.100<br>(0.700, 1.500)<br>[-0.100 - 1.900]	1.000<br>(0.500, 1.250)<br>[-1.200 - 2.800]	1.150<br>(0.600, 1.500)<br>[-0.800 - 2.500]	1.100<br>(0.650, 1.400)<br>[-0.600 - 2.300]
RWT Semantic Verbal Fluency	0.367<br>(0.878)<br>[-1.405 - 2.326]	0.704<br>(0.959)<br>[-1.227 - 2.326]	0.569<br>(0.829)<br>[-0.842 - 2.326]	0.639<br>(0.719)<br>[-0.915 - 2.326]	0.985<br>(1.097)<br>[-2.326 - 2.326]	0.944<br>(0.857)<br>[-1.227 - 2.326]
RWT Phonemic Verbal Fluency	0.364<br>(1.144)<br>[-2.054 - 2.326]	0.426<br>(0.962)<br>[-1.645 - 2.326]	0.450<br>(1.047)<br>[-2.326 - 2.326]	0.339<br>(0.927)<br>[-2.054 - 2.326]	0.677<br>(0.838)<br>[-1.036 - 2.326]	0.782<br>(0.985)<br>[-2.326 - 2.326]
TMT B/A	-0.011<br>(1.002)<br>[-2.740 - 2.100]	-0.241<br>(1.016)<br>[-2.990 - 1.480]	-0.170<br>(0.890)<br>[-2.450 - 1.440]	-0.372<br>(0.957)<br>[-2.470 - 2.110]	-0.021<br>(0.712)<br>[-1.650 - 1.270]	-0.028<br>(0.928)<br>[-2.140 - 2.070]
Stroop Reading	0.371<br>(0.720)<br>[-1.100 - 2.200]	0.415<br>(0.612)<br>[-1.000 - 1.800]	0.377<br>(0.675)<br>[-1.100 - 2.400]	0.520<br>(0.642)<br>[-0.700 - 2.000]	0.571<br>(0.666)<br>[-0.600 - 2.000]	0.572<br>(0.718)<br>[-1.100 - 2.300]
Stroop Naming	0.437<br>(0.838)<br>[-1.300 - 2.200]	0.686<br>(0.832)<br>[-0.900 - 2.100]	0.574<br>(0.870)<br>[-1.400 - 2.200]	0.523<br>(0.757)<br>[-1.200 - 2.100]	0.724<br>(0.749)<br>[-0.600 - 2.200]	0.660<br>(0.773)<br>[-0.500 - 2.300]
WMS Digit Span Backward	0.256<br>(0.959)<br>[-1.341 - 2.054]	0.152<br>(1.001)<br>[-1.645 - 1.881]	0.286<br>(0.964)<br>[-2.326 - 2.054]	0.003<br>(0.837)<br>[-1.476 - 1.751]	0.563<br>(0.934)<br>[-1.341 - 2.054]	0.362<br>(0.914)<br>[-2.054 - 1.881]
Brief Test of Attention	0.772<br>(0.253, 1.476)<br>[-0.739 - 2.326]	0.772<br>(-0.253, 1.476)<br>[-2.054 - 2.326]	0.385<br>(-0.305, 0.915)<br>[-1.881 - 2.326]	0.385<br>(-0.164, 0.915)<br>[-2.326 - 2.326]	0.468<br>(0.126, 0.915)<br>[-1.282 - 2.326]	0.620<br>(0.253, 1.476)<br>[-1.036 - 2.326]
TMT A	0.586<br>(1.014)<br>[-1.280 - 3.060]	0.910<br>(1.021)<br>[-1.380 - 2.700]	0.993<br>(1.068)<br>[-0.710 - 3.950]	0.984<br>(1.091)<br>[-0.810 - 3.650]	0.909<br>(1.216)<br>[-1.900 - 3.310]	0.924<br>(1.005)<br>[-1.100 - 3.310]
WMS Digit Span Forward	0.623<br>(0.934)<br>[-1.645 - 2.326]	0.679<br>(0.880)<br>[-1.227 - 2.326]	0.627<br>(0.948)<br>[-1.645 - 2.326]	0.352<br>(0.968)<br>[-1.645 - 2.326]	0.907<br>(0.924)<br>[-1.036 - 2.326]	0.574<br>(0.911)<br>[-1.036 - 2.054]
VLMT Learning	0.211<br>(0.907)<br>[-1.751 - 1.751]	0.162<br>(1.051)<br>[-1.751 - 1.751]	0.452<br>(0.850)<br>[-1.751 - 1.751]	0.204<br>(0.884)<br>[-1.751 - 1.751]	0.201<br>(0.987)<br>[-1.751 - 1.751]	0.562<br>(0.948)<br>[-1.751 - 1.751]
VLMT Recall I	0.189<br>(-0.755, 0.454)<br>[-1.751 - 1.751]	0.189<br>(-0.385, 0.755)<br>[-1.751 - 1.751]	0.454<br>(-0.063, 1.282)<br>[-1.440 - 1.751]	0.189<br>(-0.570, 0.605)<br>[-1.751 - 1.751]	0.454<br>(-0.755, 0.755)<br>[-1.751 - 1.751]	0.189<br>(-0.191, 0.755)<br>[-1.751 - 1.751]
VLMT Recall II	-0.063<br>(-0.674, 0.126)<br>[-1.751 - 1.311]	0.000<br>(-0.935, 0.598)<br>[-1.751 - 1.751]	0.126<br>(-0.189, 0.598)<br>[-1.751 - 1.751]	0.000<br>(-0.564, 0.598)<br>[-1.751 - 1.751]	0.063<br>(-0.674, 0.598)<br>[-1.751 - 1.751]	0.126<br>(-0.321, 0.954)<br>[-1.751 - 1.751]
VLMT Recognition	0.253<br>(-0.385, 1.058)<br>[-1.751 - 1.058]	0.253<br>(-0.842, 1.058)<br>[-1.514 - 1.058]	0.253<br>(0.253, 1.058)<br>[-1.645 - 1.058]	0.253<br>(-0.385, 1.058)<br>[-1.036 - 1.058]	0.253<br>(-0.385, 1.058)<br>[-1.440 - 1.058]	0.253<br>(0.253, 1.058)<br>[-1.440 - 1.058]
ROCFT Recall	0.965<br>(0.210, 1.310)<br>[-1.890 - 2.330]	1.170<br>(0.510, 1.890)<br>[-0.160 - 3.320]	1.170<br>(0.570, 1.930)<br>[-1.360 - 3.150]	0.850<br>(-0.125, 1.460)<br>[-1.830 - 2.500]	1.215<br>(0.700, 2.240)<br>[-1.920 - 2.990]	1.170<br>(0.410, 2.105)<br>[-1.220 - 3.640]
Benton Judgment of Line Orientation	1.080<br>(0.151, 1.080)<br>[-0.772 - 1.080]	1.080<br>(0.583, 1.080)<br>[-0.253 - 1.080]	1.080<br>(0.583, 1.080)<br>[-0.772 - 1.080]	1.080<br>(0.583, 1.080)<br>[-1.751 - 1.080]	1.080<br>(0.583, 1.080)<br>[-1.751 - 1.080]	1.080<br>(0.583, 1.080)<br>[-0.772 - 1.080]
LPS50+ Subtest 7 Rotation	0.074<br>(0.999)<br>[-2.000 - 2.000]	-0.223<br>(1.042)<br>[-2.400 - 1.600]	0.167<br>(0.976)<br>[-1.900 - 2.400]	-0.063<br>(1.060)<br>[-1.900 - 2.200]	-0.047<br>(0.993)<br>[-1.900 - 2.800]	0.269<br>(0.985)<br>[-1.800 - 2.700]
LPS50+ Subtest 11 Perception	0.100<br>(-0.600, 0.400)<br>[-1.400 - 2.000]	0.100<br>(-0.400, 0.600)<br>[-1.200 - 2.000]	0.100<br>(-0.400, 0.600)<br>[-1.200 - 2.000]	0.300<br>(-0.400, 0.850)<br>[-1.700 - 1.200]	0.600<br>(0.200, 1.000)<br>[-1.800 - 1.900]	0.600<br>(0.000, 0.900)<br>[-1.700 - 1.200]
ROCFT Copy	1.240<br>(1.240, 1.610)<br>[0.050 - 1.910]	1.240<br>(1.240, 1.910)<br>[-0.660 - 1.910]	1.240<br>(1.240, 1.910)<br>[0.760 - 1.910]	1.240<br>(1.240, 1.910)<br>[0.050 - 1.910]	1.240<br>(1.040, 1.310)<br>[-0.170 - 1.910]	1.240<br>(1.205, 1.910)<br>[0.130 - 1.910]
HAWIE Similarities	0.440<br>(0.000, 0.750)<br>[-0.650 - 2.110]	0.650<br>(0.130, 1.060)<br>[-0.650 - 2.110]	0.440<br>(-0.190, 0.860)<br>[-1.290 - 2.310]	0.650<br>(-0.030, 1.060)<br>[-0.970 - 1.690]	0.765<br>(0.140, 1.210)<br>[-0.930 - 2.030]	0.750<br>(0.135, 1.090)<br>[-0.780 - 2.000]
ACL Naming	0.000<br>(0.000, 0.000)<br>[-1.000 - 0.000]	0.000<br>(0.000, 0.000)<br>[-1.000 - 0.000]	0.000<br>(0.000, 0.000)<br>[-1.000 - 0.000]	0.000<br>(0.000, 0.000)<br>[-1.000 - 0.000]	0.000<br>(0.000, 0.000)<br>[-1.000 - 0.000]	0.000<br>(0.000, 0.000)<br>[-1.000 - 0.000]
MCI status						
no MCI	27 (64.29%)	26 (66.67%)	30 (76.92%)	29 (72.50%)	33 (86.84%)	31 (86.11%)
MCI	15 (35.71%)	13 (33.33%)	9 (23.08%)	11 (27.50%)	5 (13.16%)	5 (13.89%)

Notes. Data are mean (standard deviation) [range: minimum - maximum], median (inter quartile range) [range: minimum - maximum], or *n* (%). ACL, Aphasia Check List; HAWIE, Hamburg-Wechsler-Intelligenztest für Erwachsene; LPS50+, Leistungsprüfsystem für 50-bis 90-Jährige; MCI, mild cognitive impairment; ROCFT, Rey Osterrieth Complex Figure Test; RWT, Regensburger Wortflüssigkeitstest; TMT, Trail Making Test; VLMT, Verbaler Lern- und Merkfähigkeitstest; WMS, Wechsler Memory Scale.

Supplementary Table 4. Results of LME Models to Assess Training Effects in Exploratory Outcomes

	ANOVA of LME models			Absolute Change Score Based on EMM (SE)				timepoint × group	
				Control Group		Intervention Group		Cohen's *d* (95% Confidence Interval)	
	**timepoint**	**group**	**timepoint × group**	**t1 - t0**	**t2 - t0**	**t1 - t0**	**t2 - t0**	**t1 - t0**	**t2 - t0**
LPS50+ Subtest 4 Logical Reasoning	F(2,146.471) = 13.653, P = ≤ 0.0001, P_{FDR} = ≤ 0.0001	F(1,76.845) = 0.001, P = 0.9694, P_{FDR} = 0.9843	F(2,146.471) = 5.159, P = 0.0068, P_{FDR} = 0.0376	0.115 (0.092)	0.154 (0.092)	0.538 (0.094)	0.538 (0.094)	0.540 (0.208 – 0.872)	0.255 (-0.081 – 0.590)
Stroop Interference	F(2,148.768) = 14.753, P = ≤ 0.0001, P_{FDR} = ≤ 0.0001	F(1,80.164) = 0.154, P = 0.6954, P_{FDR} = 0.9843	F(2,148.768) = 1.027, P = 0.3605, P_{FDR} = 0.6431	0.215 (0.047)	0.174 (0.047)	0.134 (0.048)	0.134 (0.048)	-0.135 (-0.356 – 0.086)	-0.142 (-0.365 – 0.082)
RWT Semantic Verbal Fluency	F(2,151.797) = 6.273, P = 0.0024, P_{FDR} = 0.0145	F(1,80.937) = 3.574, P = 0.0623, P_{FDR} = 0.2055	F(2,151.797) = 0.092, P = 0.9118, P_{FDR} = 0.9843	0.332 (0.14)	0.197 (0.14)	0.357 (0.143)	0.357 (0.143)	0.031 (-0.457 – 0.519)	0.105 (-0.388 – 0.597)
RWT Phonemic Verbal Fluency	F(2,150.991) = 3.307, P = 0.0393, P_{FDR} = 0.1483	F(1,80.550) = 1.025, P = 0.3143, P_{FDR} = 0.6101	F(2,150.991) = 1.600, P = 0.2052, P_{FDR} = 0.4715	0.013 (0.145)	0.105 (0.145)	0.34 (0.148)	0.34 (0.148)	0.314 (-0.081 – 0.709)	0.303 (-0.096 – 0.702)
TMT B/A	F(2,150.114) = 0.283, P = 0.7538, P_{FDR} = 0.9843	F(1,78.672) = 0.002, P = 0.9652, P_{FDR} = 0.9843	F(2,150.114) = 3.277, P = 0.0405, P_{FDR} = 0.1483	-0.218 (0.172)	-0.167 (0.172)	0.348 (0.174)	0.348 (0.174)	0.571 (0.083 – 1.059)	0.517 (0.026 – 1.008)
Stroop Reading	F(2,148.948) = 0.162, P = 0.8506, P_{FDR} = 0.9843	F(1,79.901) = 1.679, P = 0.1988, P_{FDR} = 0.4715	F(2,148.948) = 0.156, P = 0.8556, P_{FDR} = 0.9843	0.003 (0.068)	-0.004 (0.068)	0.05 (0.069)	0.05 (0.069)	0.068 (-0.213 – 0.350)	0.069 (-0.215 – 0.354)
Stroop Naming	F(2,148.734) = 7.691, P = 0.0007, P_{FDR} = 0.0050	F(1,79.937) = 0.258, P = 0.6130, P_{FDR} = 0.9195	F(2,148.734) = 0.033, P = 0.9677, P_{FDR} = 0.9843	0.208 (0.072)	0.086 (0.072)	0.194 (0.073)	0.194 (0.073)	-0.017 (-0.271 – 0.238)	0.017 (-0.240 – 0.274)
WMS Digit Span Backward	F(2,150.559) = 2.679, P = 0.0719, P_{FDR} = 0.2259	F(1,79.948) = 0.057, P = 0.8116, P_{FDR} = 0.9843	F(2,150.559) = 4.912, P = 0.0086, P_{FDR} = 0.0435	-0.109 (0.141)	0.071 (0.141)	0.52 (0.144)	0.52 (0.144)	0.695 (0.255 – 1.136)	0.281 (-0.164 – 0.725)
Brief Test of Attention	F(2,151.709) = 0.049, P = 0.9524, P_{FDR} = 0.9843	F(1,80.175) = 0.082, P = 0.7750, P_{FDR} = 0.9843	F(2,151.709) = 3.111, P = 0.0474, P_{FDR} = 0.1647	-0.201 (0.173)	-0.348 (0.173)	0.157 (0.176)	0.157 (0.176)	0.380 (-0.138 – 0.898)	0.655 (0.132 – 1.178)
TMT A	F(2,149.904) = 1.549, P = 0.2158, P_{FDR} = 0.4726	F(1,79.576) = 0.378, P = 0.5402, P_{FDR} = 0.8291	F(2,149.904) = 2.364, P = 0.0976, P_{FDR} = 0.2800	0.293 (0.155)	0.42 (0.155)	-0.077 (0.157)	-0.077 (0.157)	-0.347 (-0.756 – 0.062)	-0.422 (-0.835 – -0.009)
WMS Digit Span Forward	F(2,150.615) = 4.610, P = 0.0114, P_{FDR} = 0.0537	F(1,80.105) = 0.039, P = 0.8446, P_{FDR} = 0.9843	F(2,150.615) = 3.473, P = 0.0335, P_{FDR} = 0.1476	0.036 (0.138)	-0.004 (0.138)	0.551 (0.14)	0.551 (0.14)	0.539 (0.132 – 0.945)	0.208 (-0.203 – 0.618)
VLMT Learning	F(2,149.966) = 11.017, P = ≤ 0.0001, P_{FDR} = 0.0004	F(1,80.571) = 0.003, P = 0.9568, P_{FDR} = 0.9843	F(2,149.966) = 0.171, P = 0.8433, P_{FDR} = 0.9843	-0.024 (0.108)	0.268 (0.108)	-0.042 (0.11)	-0.042 (0.11)	-0.020 (-0.361 – 0.321)	0.078 (-0.267 – 0.423)
VLMT Recall I	F(2,148.989) = 13.002, P = ≤ 0.0001, P_{FDR} = ≤ 0.0001	F(1,79.823) = 0.005, P = 0.9431, P_{FDR} = 0.9843	F(2,148.989) = 2.611, P = 0.0769, P_{FDR} = 0.2306	0.213 (0.105)	0.549 (0.105)	0.026 (0.106)	0.026 (0.106)	-0.187 (-0.482 – 0.108)	-0.343 (-0.641 – -0.045)
VLMT Recall II	F(2,149.433) = 7.658, P = 0.0007, P_{FDR} = 0.0050	F(1,80.039) = 0.920, P = 0.3405, P_{FDR} = 0.6323	F(2,149.433) = 0.121, P = 0.8858, P_{FDR} = 0.9843	0.12 (0.109)	0.328 (0.109)	0.048 (0.111)	0.048 (0.111)	-0.079 (-0.415 – 0.257)	-0.063 (-0.402 – 0.277)
VLMT Recognition	F(2,150.834) = 1.516, P = 0.2229, P_{FDR} = 0.4726	F(1,79.195) = 1.409, P = 0.2387, P_{FDR} = 0.4774	F(2,150.834) = 0.703, P = 0.4969, P_{FDR} = 0.7999	-0.162 (0.132)	0.116 (0.132)	-0.006 (0.134)	-0.006 (0.134)	0.209 (-0.287 – 0.705)	-0.086 (-0.587 – 0.414)
ROCFT Recall	F(2,148.960) = 29.898, P = ≤ 0.0001, P_{FDR} = ≤ 0.0001	F(1,79.703) = 0.000, P = 0.9984, P_{FDR} = 0.9984	F(2,148.960) = 0.163, P = 0.8498, P_{FDR} = 0.9843	0.525 (0.108)	0.454 (0.108)	0.54 (0.11)	0.54 (0.11)	0.015 (-0.297 – 0.328)	0.086 (-0.230 – 0.402)
Benton Judgment of Line Orientation	F(2,148.064) = 1.591, P = 0.2072, P_{FDR} = 0.4715	F(1,78.572) = 0.409, P = 0.5246, P_{FDR} = 0.8243	F(2,148.064) = 1.676, P = 0.1906, P_{FDR} = 0.4715	0.125 (0.068)	0.07 (0.068)	-0.014 (0.07)	-0.014 (0.07)	-0.223 (-0.532 – 0.086)	0.052 (-0.261 – 0.364)
LPS50+ Subtest 7 Rotation	F(2,150.276) = 10.142, P = ≤ 0.0001, P_{FDR} = 0.0007	F(1,80.931) = 0.038, P = 0.8456, P_{FDR} = 0.9843	F(2,150.276) = 1.845, P = 0.1616, P_{FDR} = 0.4443	-0.294 (0.114)	0.11 (0.114)	0.001 (0.116)	0.001 (0.116)	0.288 (-0.025 – 0.601)	0.226 (-0.090 – 0.542)
LPS50+ Subtest 11 Perception	F(2,149.280) = 15.051, P = ≤ 0.0001, P_{FDR} = ≤ 0.0001	F(1,80.245) = 4.338, P = 0.0405, P_{FDR} = 0.1483	F(2,149.280) = 0.710, P = 0.4932, P_{FDR} = 0.7999	0.211 (0.073)	0.191 (0.073)	0.33 (0.074)	0.33 (0.074)	0.162 (-0.115 – 0.440)	0.042 (-0.238 – 0.323)
ROCFT Copy	F(2,142.724) = 1.796, P = 0.1698, P_{FDR} = 0.4482	F(1,72.310) = 0.235, P = 0.6290, P_{FDR} = 0.9225	F(2,142.724) = 0.818, P = 0.4435, P_{FDR} = 0.7505	0.104 (0.072)	-0.06 (0.072)	0.006 (0.073)	0.006 (0.073)	-0.202 (-0.621 – 0.217)	0.063 (-0.360 – 0.486)
HAWIE Similarities	F(2,149.016) = 6.774, P = 0.0015, P_{FDR} = 0.0101	F(1,79.617) = 1.469, P = 0.2292, P_{FDR} = 0.4726	F(2,149.016) = 1.072, P = 0.3449, P_{FDR} = 0.6323	0.208 (0.079)	0.023 (0.079)	0.209 (0.081)	0.209 (0.081)	0.000 (-0.341 – 0.342)	0.225 (-0.121 – 0.570)
ACL Naming	F(2,228.000) = 0.170, P = 0.8441, P_{FDR} = 0.9843	F(1,228.000) = 0.006, P = 0.9390, P_{FDR} = 0.9843	F(2,228.000) = 0.819, P = 0.4424, P_{FDR} = 0.7505	-0.029 (0.049)	0.022 (0.049)	0.049 (0.05)	0.049 (0.05)	0.324 (-0.256 – 0.904)	-0.011 (-0.594 – 0.573)

Notes. ACL, Aphasia Check List; ANOVA, analysis of variance; EMM, estimated marginal means; HAWIE, Hamburg-Wechsler-Intelligenztest für Erwachsene; LME, linear mixed effects; LPS50+, Leistungsprüfsystem für 50-bis 90-Jährige; MCI, mild cognitive impairment; ROCFT, Rey Osterrieth Complex Figure Test; RWT, Regensburger Wortflüssigkeitstest; SE, standard error; TMT, Trail Making Test; VLMT, Verbaler Lern- und Merkfähigkeitstest; WMS, Wechsler Memory Scale.
